# Supplementary material for: Infection of Brain Pericytes Underlying Neuropathology of COVID-19 Patients
Source: Int J Mol Sci. 2021 Oct 27;22(21):11622. doi: 10.3390/ijms222111622 (PMC8583965; doi:10.3390/ijms222111622)
Supplement: Supplementary file 1 [file ijms-22-11622-s001.zip › ijms-1418151-supplementary.pdf]

Supplementary Table S1 Characteristics of the COVID-19 patients and relative controls (brain and placenta IHC/mIHC study, Lund University)

Patient ID	Age (years)	Gender	ACE2	Vascular histopathological evaluation	Neurological assessment
COVID-19 1	67	M	High	Small vessel disease, signs of cerebral hypoperfusion	Unconscious >4 weeks before death
COVID-19 2	54	M	High	Mild but general edema of CNS	Unconscious >4 weeks before death
COVID-19 3	72	M	Moderate	No remarks	Unconscious >1 week before death
COVID-19 4	80	M	Not detected	No remarks	Alert except for two days of confusion
COVID-19 5	57	M	Not detected	Perivascular lymphocytic infiltration/vascular thrombosis	Alert until death
COVID-19 6	75	F	Not detected	No remarks	Alert until death
Control 1	73	M	Not detected	No remarks	No remarks, Alert until death
Control 2	68	F	High	Hypertensive angiopathy and microthrombosis	Decline before death
Control 3	72	F	Low	Severe vascular/ischemic lesions	No remarks
Control 4	82	F	High	Progressive multifocal leukoencephalopathy	No remarks
Control 5	37	M	Low	No remarks	Alert until death
Control 6	68	F	Low	Small vessel disease, microbleeds, stroke in brainstem	No remarks
Control 7	29	M	Low	No remarks	No remarks
COVID-19 placenta	27	F	High	Not applicable	Not applicable
Control placenta	28	F	High	Not applicable	Not applicable

Supplementary Table S2 Characteristics of the COVID-19 patients and relative controls (CSF study, Sahlgrenska University hospital, Gothenburg)

Patient ID	Age (years)	Gender	PDGFRβ (pg/ml)	Interval[a] (days)	Neurological assessment
COVID-19 CSF 1	84	F	1065	10	Confusion
COVID-19 CSF 2	70	M	459	6	Confusion, fatigue, delayed response to questions
COVID-19 CSF 3	62	M	976	15	Confusion, personality changes, delayed response to questions, disorientation
COVID-19 CSF 4	64	M	275	7	Altered consciousness/somnolence, neck stiffness, photophobia
COVID-19 CSF 5	42	M	602	12	Exhaustion, dysgeusia
COVID-19 CSF 6	72	M	772	12	Confusion, delayed response to questions
COVID-19 CSF 7	59	F	678	13	Confusion, fatigue, dysarthria, myoclonus
COVID-19 CSF 8	78	F	611	10	Confusion
Control CSF 1	72	F	1308	Not applicable	Not applicable
Control CSF 2	76	M	1938	Not applicable	Not applicable
Control CSF 3	56	M	705	Not applicable	Not applicable
Control CSF 4	72	M	738	Not applicable	Not applicable
Control CSF 5	47	M	1246	Not applicable	Not applicable
Control CSF 6	73	M	1051	Not applicable	Not applicable
Control CSF 7	59	F	905	Not applicable	Not applicable
Control CSF 8	67	F	941	Not applicable	Not applicable

[a]Days between the initial report of COVID-19 symptoms and the lumbar puncture.

Supplementary Table S3 Antibody list

Target	Manufacturer	Article number	Host	Reactivity	Clonality	Dilution	HIER[a]	Application
ACE2	Abcam	Ab108252	Rabbit	Human, Mouse, Rat	Monoclonal EPR4435[2]	1:100	Citrate buffer (pH6)	IHC/mIHC
ACE2	R&D	MAB933	Mouse	Human, Hamster	Monoclonal 171606	1:50	Tris-EDTA buffer (pH9)	IHC
CD4	Akoya Biosciences	Kit: OP7TL4001KT		Human		70 ng/ml	Tris-EDTA buffer (pH9)	mIHC
CD8	Akoya Biosciences	Kit: OP7TL4001KT		Human		120 ng/ml	Tris-EDTA buffer (pH9)	mIHC
CD20	Akoya Biosciences	Kit: OP7TL4001KT		Human		100 ng/ml	Citrate buffer (pH6)	mIHC
CD31	Cell Signaling Technology	3528S	Mouse	Human	Monoclonal 89C2	1:1000	Citrate buffer (pH6)	mIHC
CD34	Novus Biologicals	NBP2-32932	Mouse	Human, Primate	Monoclonal QBEnd/10	1:1000	Citrate buffer (pH6)	mIHC
CD68	Akoya Biosciences	Kit: OP7TL4001KT		Human		15 ng/ml	Citrate buffer (pH6)	mIHC
dsRNA	SCICONS	10010200	Mouse	Virus	Monoclonal J2	1:500	Tris-EDTA buffer (pH9)	IHC/mIHC
Fibrinogen	Abcam	Ab189490	Rabbit	Human, Mouse, Rat	Monoclonal EPR18145-84	1:4000	Tris-EDTA buffer (pH9)	IHC/mIHC
FOXP3	Akoya Biosciences	Kit: OP7TL4001KT		Human		700 ng/ml	Citrate buffer (pH6)	mIHC
GFAP	Atlas Antibodies	HPA056030	Rabbit	Human, Mouse		1:2500	Citrate buffer (pH6)	IHC/mIHC
NeuN/RBFOX3	Atlas Antibodies	HPA030790	Rabbit	Human	Polyclonal	1:200	Citrate buffer (pH6)	mIHC
pan-CK	Akoya Biosciences	Kit: OP7TL4001KT		Human		400 ng/ml	Citrate buffer (pH6)	mIHC
PDGFRβ	Cell Signaling Technology	3169S	Rabbit	Human, Mouse, Rat	Monoclonal 28E1	1:75	Tris-EDTA buffer (pH9)	mIHC
SARS-CoV nucleocapsid	Novus Biologicals	NB100-56576SS	Rabbit	Virus	Polyclonal	1:100	Citrate buffer (pH6)	IHC/mIHC
SARS-CoV-2 nucleocapsid	Sino Biologicals	40143-019	Rabbit	Virus	Monoclonal 019	1:100	Citrate buffer (pH6)	IHC
SARS-CoV-2 spike	GeneTex	GTX135356	Rabbit	Virus	Polyclonal	1:100	Citrate buffer (pH6)	IHC/mIHC
SARS-CoV-2 spike	Sino Biological	40150-R007	Rabbit	Virus	Monoclonal 007	1:100	Citrate buffer (pH6)	IHC/mIHC

[a]HIER: heat-induced epitope retrieval

Supplementary Table S4 Multiplex IHC panels

Panel	Position 1	Position 2	Position 3	Position 4	Position 5	Position 6
**Neurovascular unit (Fig.1E)**						
HIER[a]/Stripping	pH9	pH6	pH6			
Blocking		10 min, RT				
Primary antibody	PDGFRβ	ACE2	CD31			
Dilution	1:75	1:100	1:1000			
Incubation (time, temperature)		30 min, RT				
OPAL	OPAL 480	OPAL 620	OPAL 690			
Dilution	1:100	1:100	1:100			
Incubation (time, temperature)		10 min, RT				

Panel	Position 1	Position 2	Position 3	Position 4	Position 5	Position 6
**Placenta (Fig. 2B)**						
HIER/Stripping	pH9	pH9	pH6	pH6	pH6	pH6
Blocking			10 min, RT			
Primary antibody	dsRNA	PDGFRβ	ACE2	Pan-CK	CD68	CD31
Dilution	1:500	1:75	1:100			1:1000
Incubation (time, temperature)			30 min, RT			
OPAL	OPAL 480	OPAL 520	OPAL 570	OPAL 620	OPAL 690	OPAL 780
Dilution	1:100	1:50	1:50	1:50	1:50	1:25
Incubation (time, temperature)			10 min, RT			

Panel	Position 1	Position 2	Position 3	Position 4	Position 5	Position 6
**Immunology (Fig. 2E)**						
HIER/Stripping	pH9	pH9	pH6	pH6	pH6	pH6
Blocking			10 min, RT			
Primary antibody	CD4	CD8	CD20	FOXP3	CD68	CD34
Dilution	70 ng/ml	120 ng/ml	100 ng/ml	700 mg/ml	15 ng/ml	1:1000
Incubation (time, temperature)			30 min, RT			
OPAL	OPAL 520	OPAL 570	OPAL 540	OPAL 620	OPAL 650	OPAL 690
Dilution	1:50	1:50	1:50	1:50	1:50	1:50
Incubation (time, temperature)			10 min, RT			

Panel	Position 1	Position 2	Position 3	Position 4	Position 5	Position 6
**Fibrinogen (Fig. 3A)**						
HIER/Stripping	pH9	pH9	pH9	pH6	pH6	pH6
Blocking			10 min, RT			
Primary antibody	dsRNA	PDGFRβ	Fibrinogen	ACE2	NeuN	CD34
Dilution	1:500	1:75	1:4000	1:100	1:200	1:1000
Incubation (time, temperature)			30 min, RT			
OPAL	OPAL 480	OPAL 520	OPAL 570	OPAL 620	OPAL 690	OPAL 780
Dilution	1:100	1:50	1:50	1:50	1:50	1:25
Incubation (time, temperature)			10 min, RT			

Panel	Position 1	Position 2	Position 3	Position 4	Position 5	Position 6
**Astrocytes (Fig. 3B)**						
HIER/Stripping	pH9	pH9	pH6	pH6	pH6	pH6
Blocking			10 min, RT			
Primary antibody	PDGFRβ	Fibrinogen	ACE2	GFAP	CoV-2 spike	CD34
Dilution	1:75	1:4000	1:100	1:2500	1:100	1:1000
Incubation (time, temperature)			30 min, RT			
OPAL	OPAL 520	OPAL 570	OPAL 540	OPAL 620	OPAL 650	OPAL 690
Dilution	1:50	1:50	1:50	1:50	1:50	1:50
Incubation (time, temperature)			10 min, RT			

[a]HIER: heat-induced epitope retrieval

Bocci, Oudenaarden et al., Fig. S1

A

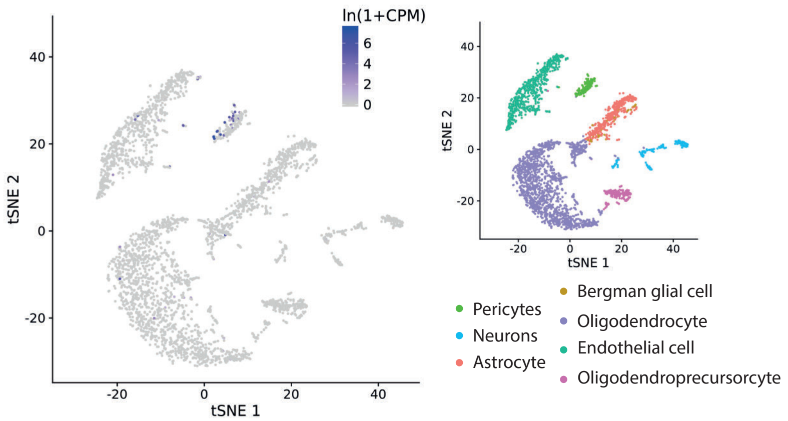

B

Gene name	Pericyte expression	Other cell expression	Fold pericyte enrichment
Kcnj8	1670.21	87.51	19.09
Pdgfrb	2525.16	369.12	6.81
Cd248	404.39	75.13	5.38
Ace2	273.26	54.83	4.98
Rgs5	2000.1	463.86	4.31

C

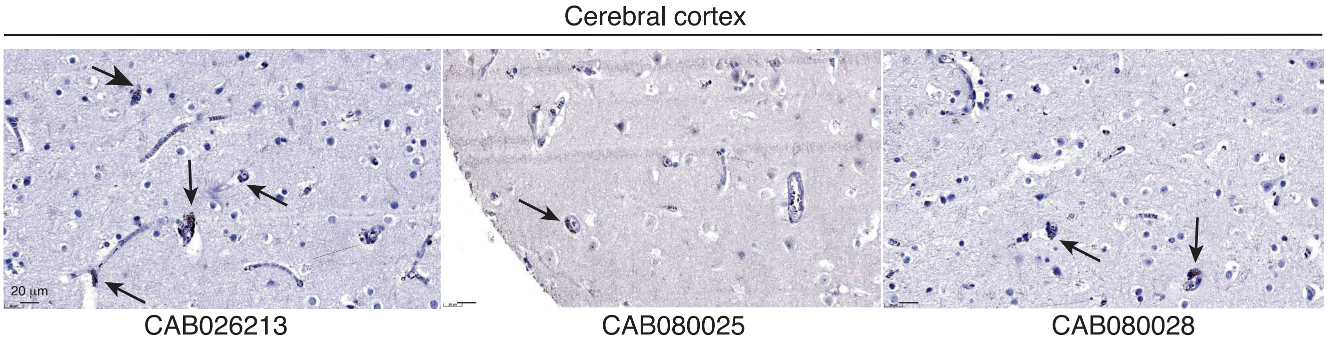

D

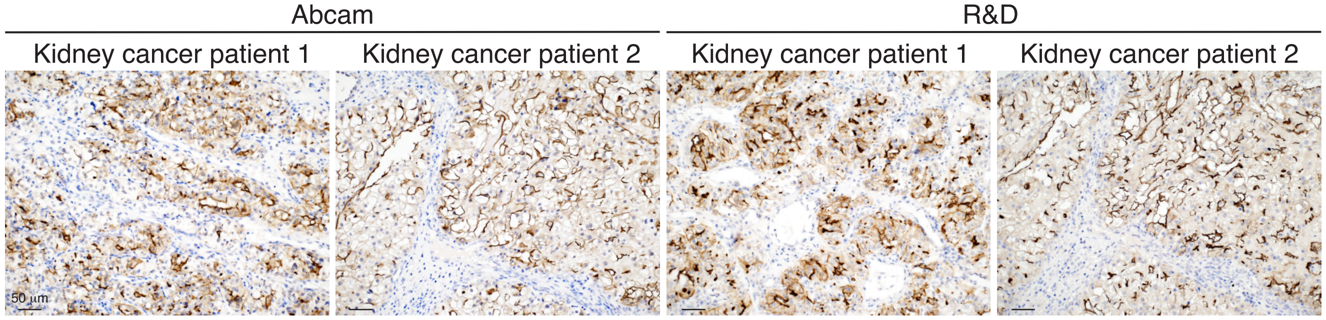

E

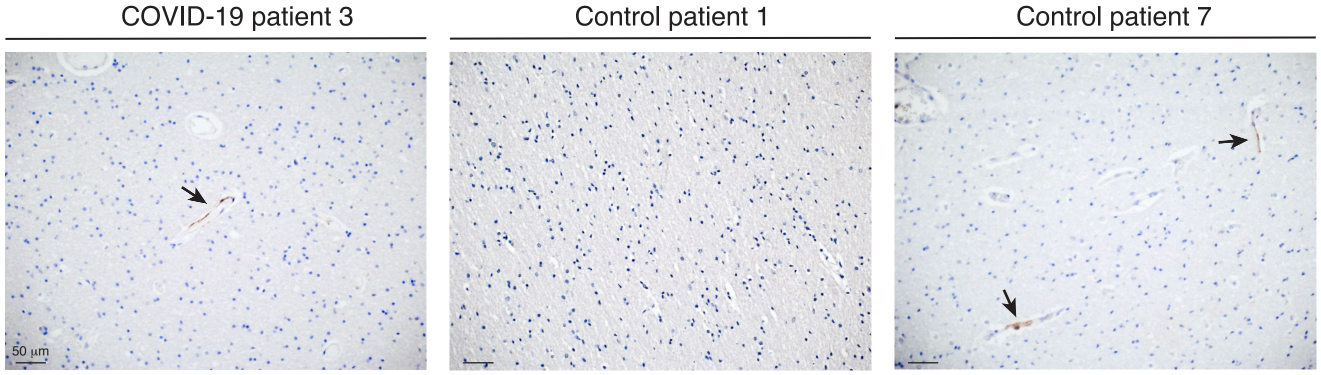

F

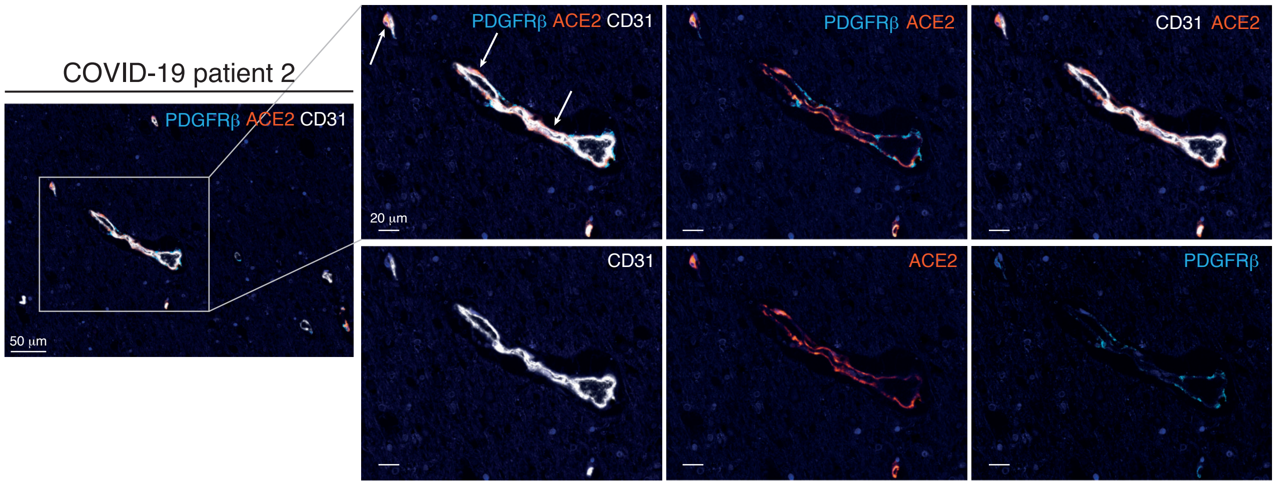

Bocci, Oudenaarden et al., Fig. S2

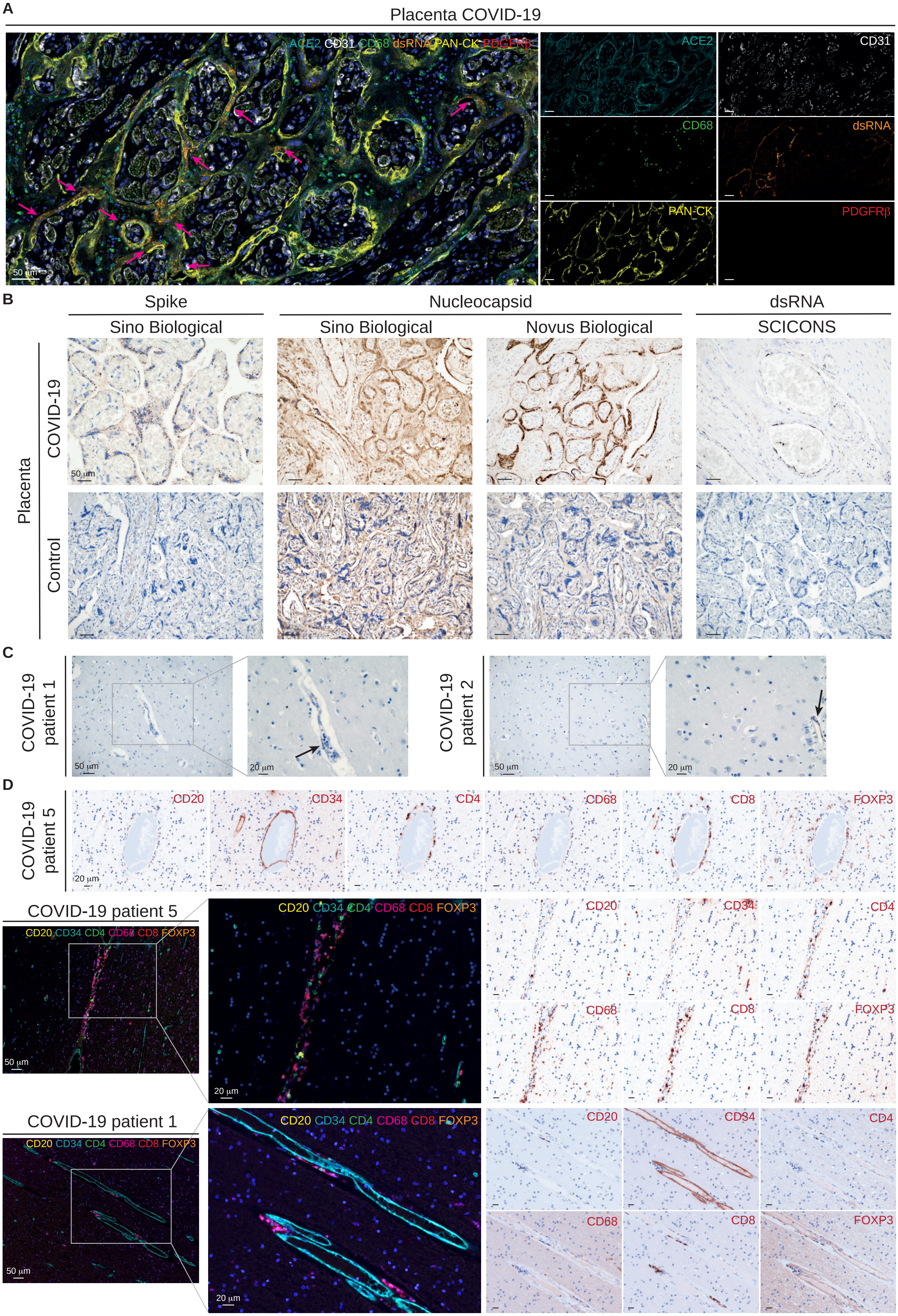

Bocci, Oudenaarden et al., Fig. S3

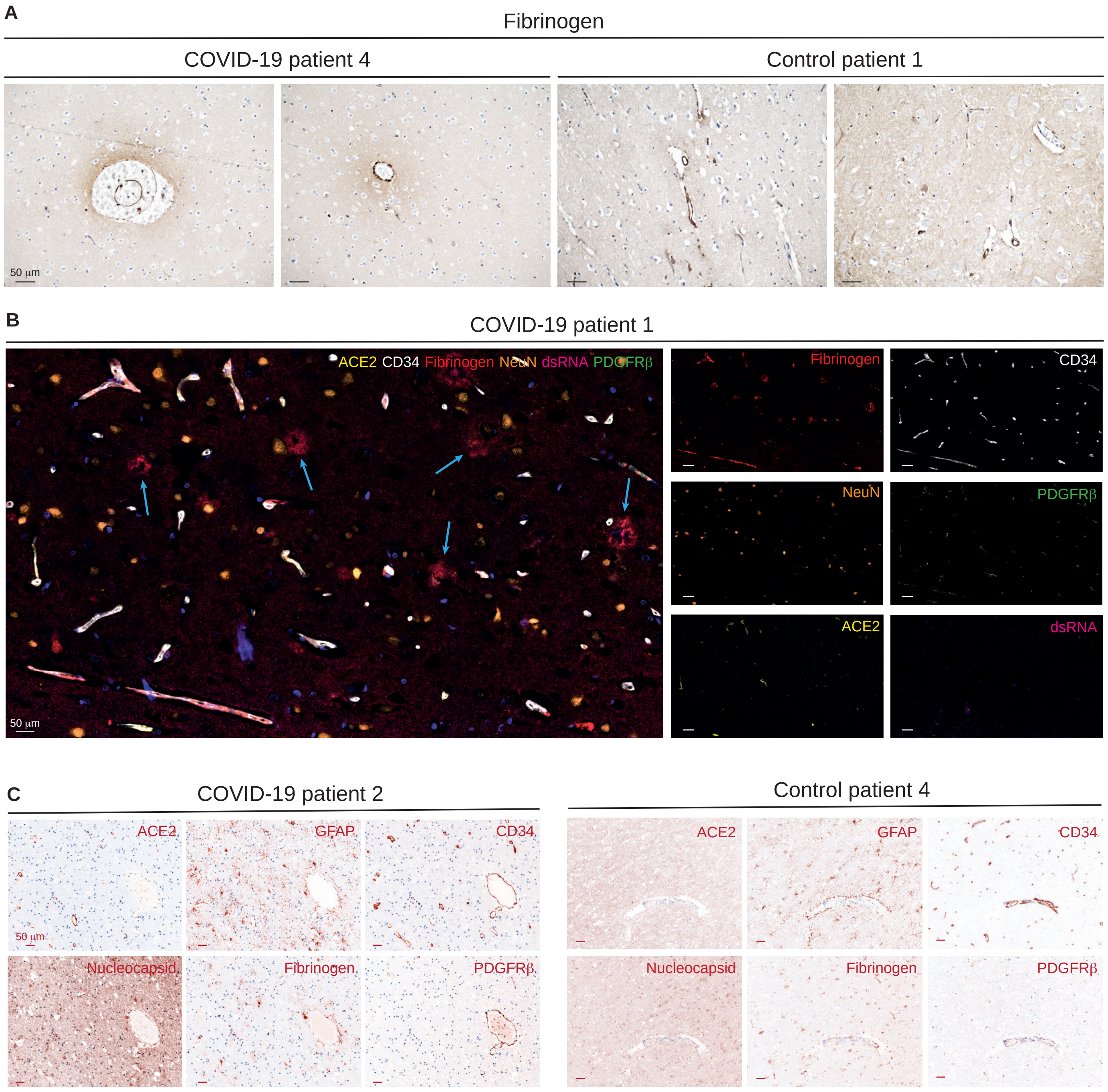

Supplementary figure legends

Figure S1. ACE2 expression in mouse and human vasculature. (**A**) Feature plot of *Ace2* expression in seven distinct color-coded cell ontology classes (tissue reference: brain non-myeloid) from the *Tabula muris* compendium. (**B**) Enrichment for *Ace2* in mouse brain pericytes compared with other brain cell types based on the Betsholtz Atlas. (**C**) representative IHC staining of ACE2 in the cerebral cortex of three patients (and three antibodies) from the Human Protein Atlas initiative. Cell nuclei are counterstained with hematoxylin (blue). The black arrows indicate $ACE2^{+}$ areas. (**D**) Representative IHC staining of ACE2 with two distinct antibodies in two different biopsies of human renal cell carcinoma. Cell nuclei are counterstained with hematoxylin (blue). (**E**) Representative IHC staining of peri-vascular ACE2 in the frontal cortex of one COVID-19 patients and two control individuals. Cell nuclei are counterstained with hematoxylin (blue). The black arrows indicate the chromogenic deposition of the DAB substrate. (**F**) Additional field of the 4-plex mIHC staining of the frontal cortex of the COVID-19 patient in Figure 1E. The final composite image depicts CD31 (endothelial cells, white), PDGFRβ (pericytes, cyan), and ACE2 (orange). Cell nuclei are counterstained with DAPI (blue). In the inlets, the white arrows indicate ACE-positive signal in the abluminal side of CD31. The intensity of each individual OPAL fluorophore, and the combined PDGFRβ/ACE2 and CD31/ACE2 overlays are presented.

Figure S2. Detection of viral dsRNA in human placenta and immune cell localization in human brain. (**A**) Additional field of the 7-plex mIHC staining panel of placental tissue infected with SARS-CoV-2 presented in Figure 2D. The magenta arrows indicate accumulation of viral dsRNA in correspondence of the ACE2-positive areas by the specialized epithelial layer of syncytiotrophoblast in the placenta. The intensity of each OPAL fluorophore is further presented in individual photomicrographs. (**B**) Immunohistochemical detection of viral components and dsRNA in a COVID-19-infected placenta and in a normal placental specimen. Cell nuclei are counterstained with hematoxylin (blue). (**C**) Immunohistochemical detection of viral dsRNA in two COVID-19-infected brains. Cell nuclei are counterstained with hematoxylin (blue). (**D**) Multiplex IHC fields of the peri-vascular immune cell infiltration in the frontal cortex of two COVID-19 patients. The antibody panel was designed for the concomitant detection of CD34 (endothelium) and five immune cell markers: CD4 (T helper cells), CD8 (cytotoxic T lymphocytes), CD20 (B cells), CD68 (macrophages), and FOXP3 (regulatory T cells). Individual OPAL intensities are displayed as “pathology view”, in which the fluorescent signal is transformed into a digitalized chromogenic DAB-like deposit.

Figure S3. Visualization of fibrinogen in brain tissue of COVID-19 patients. (**A**) Representative IHC of fibrinogen staining in the brain of a COVID-19 patients and in control tissue. Cell nuclei are counterstained with hematoxylin (blue). (**B**) Additional field of the composite mIHC in the COVID-19 patient presented in Figure 3A. The fields depict the neurovascular unit (CD34, PDGFRβ, and ACE2), fibrinogen, viral dsRNA, and neurons. The cyan arrows indicate fibrinogen leakage The intensity of each OPAL fluorophore is further presented in individual photomicrographs. (**C**) Individual OPAL intensities of the two panels presented in Figure 3A are displayed as "pathology view", in which the fluorescent signal is transformed into a digitalized chromogenic DAB-like deposit.
